# geneHapR: an R package for gene haplotypic statistics and visualization

**DOI:** 10.1186/s12859-023-05318-9

**Published:** 2023-05-15

**Authors:** Renliang Zhang, Guanqing Jia, Xianmin Diao

**Affiliations:** Institute of Crop Sciences, Chinese Academy of Agricultural Sciences, Beijing, China

**Keywords:** geneHapR, R, Haplotype identification, Visualization, Network, LD-block

## Abstract

**Background:**

Together with application of next-generation sequencing technologies and increased accumulation of genomic variation data in different organism species, an opportunity for effectively identification of superior alleles of functional genes to facilitate marker-assisted selection is emerging, and the clarification of haplotypes of functional genes is becoming an essential target in recent study works.

**Results:**

In this paper, we describe an R package ‘geneHapR’ developed for haplotypes identification, statistics and visualization analysis of candidate genes. This package could integrate genotype data, genomic annotating information and phenotypic variation data to clarify genotype variations, evolutionary-ship, and morphological effects among haplotypes through variants visualization, network construction and phenotypic comparison. ‘geneHapR’ also provides functions for Linkage Disequilibrium block analysis and visualizing of haplotypes geo-distribution.

**Conclusions:**

The R package ‘geneHapR’ provided an easy-to-use tool for haplotype identification, statistic and visualization for candidate gene and will provide useful clues for gene functional dissection and molecular-assistant pyramiding of beneficial alleles of functional locus in future breeding programs.

**Supplementary Information:**

The online version contains supplementary material available at 10.1186/s12859-023-05318-9.

## Introduction

Haplotype is a linear combination of variants in specific genomic region. Identification of superior haplotypes of functional genes are essential for developing markers for effective breeding work [1] and dissecting casual variants of target morphological and physiological traits [2–4]. Recent advances of next-generation sequencing (NGS) technologies and accumulation of genomic and phenotypic variation data make it possible for haplotype detection of nearly all effective locus applied in future breeding programs. Therefore, an efficiency tool is urgent for connecting of bio-data and breeders or molecular genetics researchers, due to haplotype analysis is becoming essential part of high impacted researches reported in recent years that focus on gene functional validation and application in plants [5, 6], animals [3, 7] and human beings [8, 9].

Haplotypes of target genes could be identified from genomic variations using many programs, including pegas [10], DnaSP [11] and CandiHap [12]. However, the visualized summarization of haplotypic variations of given locus was still challenging for current programs. For instance: Linkage disequilibrium block (LD-block) analysis and visualization is supported by HaploView [13], and haplotype network calculation and visualization are specifically supported by CandiHap and Network, while group information analysis was not supported by CandiHap. To date, a thoroughly haplotype analysis and thence summarized visualization require two or more programs, which are time consuming due to data conversion and results migrating.

Marker-assisted breeding in crop species relies on pyramiding of superior haplotypes related to target traits [14]. Superior haplotypes could be identified through phenotype comparison analysis and visualized by programs of SPSS, Excel or by R packages like haplo.stats [15] and ggplot2 [16]. However, plotting of evolutionary relationship of variants, geo-distribution and LD-block of each haplotypes requires more professional programs, which were challenging for preliminary users to grasp. As a consequence, haplotypic identification and visualization analysis cannot yet be accomplished by single accessible program at present, and it is a huge challenge for breeders and molecular biologists who are not familiar with complicated command line programs.

Moreover, screening of superior haplotypes from huge amount bio-data generated by next-generation sequencing platform were also severely restricted in current programs such as: pegas [10] and DnaSP [11], or required complicated data format conversion process which impact the efficiency of haplotype identification analysis, such as: the pipeline applied in CandiHap [12]. Hence, an easy-to-use and efficient tool for haplotype identification, statistics and visualization analysis based on NGS platform is essential for future research programs.

Here, we introduced geneHapR, a toolkit developed in R language, for haplotypic statistics of functional genes including haplotype identification, morphological effects analysis and results visualization for researchers.

## Implementation

**Fig. 1:**
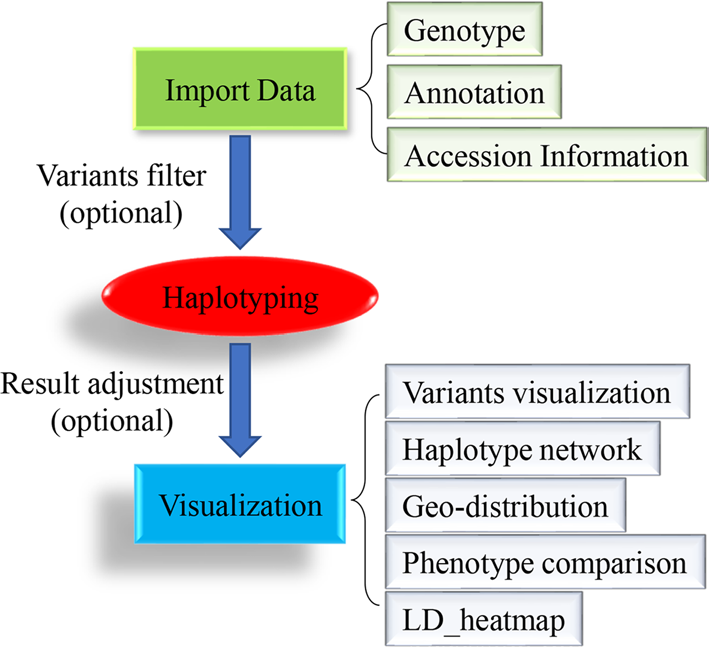
The workflow of geneHapR. There are three essential and two optional steps for haplotype identification using geneHapR. The required input datasets are genotype, annotations and accession information. And the output visualizing results include haplotype genotypes, haplotype network, geo-distribution, phenotype comparisons and LD-blocks

The geneHapR is developed in R language and three essential steps were required for haplotype analysis, including data importing, haplotype identification and haplotype visualization (Fig. 1). In addition, two optional steps, including filter of variants and adjustment of haplotype results were also realizable in geneHapR.

### Format of imported datasets and relevant functions

The geneHapR requires three types of imported data: genotype, annotation, and accession information. The first type is genotype data that could be retrieved from published variants database, or obtained by Sanger sequencing or extracted from variants calling results of next generation sequencing or micro-array. Sequences were usually stored in FASTA format file and variants were usually stored in VCF, P.link or HapMap format files. Variants in published database could also be retrieved as above format.

The second type of imported data is annotation files. Annotation files of sequenced species could be retrieved as GFF/GFF3 format from published database, such as: Phytozome [17]. For species that have not been included in published database, annotation files could be prepared in a custom BED4/6 format. Contents of each column in the custom BED4/6 are complied with the definition at Genome Browser FAQ (http://genome.ucsc.edu/FAQ/FAQformat.html#format1). The columns contents of BED6 format are: (1) chromosome name, (2) chromosome start, (3) chromosome end, (4) name, (5) score and (6) strand. In custom BED6 format, contents of the fourth column were custom as name and type, which were separated by a space. For example, “LOC_Os07g15770.1 CDS” indicates the CDS of LOC_Os07g15770.1. And the BED4 format contains the first 4 columns of custom BED6 format and DNA strands was set as positive by default.

The third type of imported data is accession/individual information, including phenotypic data, individual group/category and geographic coordinates including longitude and latitude information. An example of accession information was showed in Table 1. The accession names were defined in the first column and followed by other information (Subpopulation for accession category, longitude and latitude for geographic coordinates, grain length and grain width for phenotypes).

**Table 1:** An example of detailed accession information

ID	Subpopulation	Longitude	Latitude	Grain length	Grain width
C001	Indica	121	14.6	8.50	2.90
C002	Intermediate	121	14.6	10.20	2.63
C003	Japonica	51.3	35.45	8.75	3.32
C004	Japonica	116.28	39.54	7.83	3.22
C005	Japonica	121	14.6	10.47	3.00
C006	Indica	116.28	39.54	8.10	2.47

All required data could be imported by functions listed in Table 2. The “import_vcf()” function was used for importation of VCF file based on the vcfR package [18]. The “import_seqs()” function was used for importation of sequences in Fasta format based on the Biostrings package. And the annotation files in GFF and BED format could be imported using “import_gff()” or “import_bed()” command based on the rtracklayer package [19].

**Table 2:** The required format of datasets and import functions of geneHapR

Datasets	Input file format	Import function
Genotype (necessary)	VCF: *.vcf, *.vcf.gz; FASTA: *.fa, *.fasta; P.link: (*.ped & *.map); hmp: *.hmp; table: *.txt, *.csv	import_vcf(); import_seqs(); import_plink.pedmap(); import_hmp(); read.table(), read.csv()
Annotation (optional)	GFF: *.gff, *.gff3, BED4/BED6: *.bed	import_gff(); import_bed()
Accession information (optional)	table: *.txt, *.csv	read.table(), read.csv()

### Variants extraction

Usually, genomic variants of interested genes could be retrieved as VCF, P.link HapMap or table format from target database. However, for species with no variants database, users can easily extract interested genomic regions from the original variants file with functions like “filter*()”. For example, the “filter_VCF()” function provides a convenient way to extract variants from VCF document. There were three modes for variants extraction, (1) by position, require a chromosome name and boundary of target region; (2) by region type, require annotation and specified a type for extraction; (3) by both of position and region type.

However, there is a bottleneck for extracting variants from huge documents using personal computer, due to R needs to import the entire dataset. So, we complimented functions looks like “filterLarge*()” for variants extraction from huge documents, such as: “filterLargeVCF()” for huge VCF file, and “filterLargeP.link()” for huge P.link document.

### Haplotype identification

Haplotype was identified by functions like “*2hap()” listed in Table 3, eg.: “vcf2hap()” for genomic data in VCF format. Individuals containing missing or heterozygotes loci could be eliminated by setting parameter “na_drop” and “hetero_remove” as “TRUE”.

**Table 3:** Functions for haplotype identification

Source data format	Function
VCF	vcf2hap()
P.link(ped&map)	plink.pedmap2hap()
Fasta	seqs2hap()
HapMap	hmp2hap()
Table	table2hap()

There are two main steps for haplotype identification. Firstly, determine genotype of each accession and deal with accessions with missing genotypes; secondly, designate haplotypic names to all genotypes.

### The format of haplotype results in geneHapR and adjustment

In gene haplotype results, most users prefer nucleotide coordinates start from start codon. However, the coordinate of variants in next-generation sequencing database were usually based on chromosome. Thus, we complimented “hapSetATGas0()” and “gffSetATGas0()” functions for conveniently conversion of coordinate in gene haplotype and annotation files, respectively. Additionally, rare haplotypes could be eliminated by “filter_hap()” function.

Furthermore, haplotype results could be converted into standard format for other software by functions like hap2*(), for example, function “hap2DNAbin()” and “hap2hmp()” can convert haplotype result into DNAbin object or HapMap format.

**Fig. 2:**
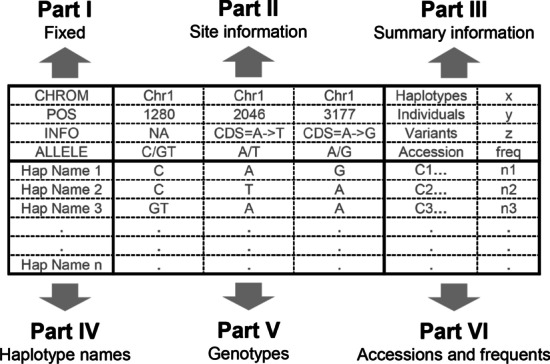
The schematic diagram of *hapResult* and *hapSummary*. The *hapResult* and *hapSummary* could be divide into six parts. Part I was fixed and consists of “CHROM”, “POS”, “INFO” and “ALLELE”, indicates the contents type of each line in Part II. Part II contains four lines storing variants information. There is several summary information of current object in part III. The part IV, V and VI includes haplotypes related information, the haplotype names were included in part IV, genotypes were included in part V, accession names and frequents were included in part VI

Haplotype results can be saved and re-imported for re-analysis using “write_hap()” and “import_hap()” function, respectively. For haplotype results, we defined *hapResult* and *hapSummary* class in R, both included a matrix storing the haplotype genotypes and other related information. For clear demonstration, the two matrices in *hapResult* and *hapSummary* were divided into six parts as shown in Fig. 2.

The part I was fixed as “CHROM”, “POS”, “INFO” and “ALLELE” indicates the contents type of each line in Part II, which contains four lines storing variants information including chromosome, position, information of each variant and alleles, respectively. There is several summary information of current object in part III, like the total number of haplotypes, individuals and variants. The part IV, V and VI includes haplotypes related information, like haplotype names in part IV, genotypes in part V, accession names and frequents in part VI. The difference between *hapResult* and *hapSummary* is that each row of part IV–VI represents single individual in *hapResult*; but represents a haplotype in *hapSummary* and the frequent column only exists in *hapSummary*.

### Visualization of haplotype results and statistics

In visualization functions, we introduced algorithms for network construction, LD-block calculation. And we personalized visualizing functions from pegas for haplotype network, from maps for geographic distribution, and from genetics for LD-block analysis. With personalized functions users can easily visualize their haplotypes results and statistics without complex format conversion.

#### Haplotype visualization

Firstly, haplotype variants could be visualized as a table-liked figure by “plotHapTable()” function. The complementation of this function is based on the ggplot2 package. This function takes the haplotype result as input and generates an R object of ggplot class. Hence, theme of the visualization could be personalized with ggplot2 package. For visualizing the relative variant positions we presented “displayVarOnGeneModel()” function which takes the annotation and haplotype result as inputs. With this function, users can easily display the variants and coordinates upon the gene schematic diagram.

#### Haplotype evolutionary ship

For illustrating relationships between haplotypes, “get_hapNet()” function was developed for haplotype network construction and “plot_hapNet()” function was developed for further visualization. In the haplotype network, each circle represents a haplotype and the size indicates individual number, and dots/short lines along the links represents variants between the haplotypes.

#### Haplotypes geographical distribution

Many traits are influenced by geographical location, due to differences of day length, rainfall, and altitude conditions. The “hapDistribution()” function was developed for demonstrating distribution of major haplotypes across global/region map. The “symbol.size” was designed as circle size controller, and “show.label” controls exhibition of the accession number in each location (circle). Although there is no limitation of haplotype display in this function, for a better readability, we suggest no more than 4 major haplotypes included in this analysis.

#### Identification of superior haplotype

To identify superior haplotype, “hapVsPheno()” and “hapVsPhenos()” function were developed for identification of phenotypic differences between haplotypes. The outliers (bigger than Q3 + 3*(Q3 – Q1) or less than Q1 – 3*(Q3 − Q1)) were removed before the calculation of significance by default. And the significances and *p* values were marked upon corresponded comparison. The rare haplotype with group member less than 5 won’t be analyzed by default.

#### Linkage disequilibrium (LD) analysis

After identification and confirmation of main effect genomic variants contributing to target traits, DNA markers need to be developed for molecular assisted selection. The genomic variant itself is an ideal choice for marker development. Moreover, linked genomic variations could also be used for marker development. The linkage disequilibrium analysis could be conducted by “plot_LDheatmap()” function and will be helpful for screening of closely linked variants.

## Results

### Data preparation of a grain size regulating gene in rice

For demonstrating the usage of geneHapR, the genotype and phenotype data and annotation of a grain size regulating gene *OsGHD7* [20, 21] were retrieved from Rice Variation Map [22] and Rice Functional Genomics and Breeding [23] and Rice Genome Annotation Project [24], respectively.  The R script used for haplotype identification and visualzation of *OsGHD7 *could be found in additional file 1.

### Haplotype statistics and visualization of *OsGHD7*

Firstly, genotype (Additional file 2), annotation (Additional file 3), phenotype (Additional file 4), and accession information (Additional file 5) were imported by functions listed in Table 1. And then haplotype of *OsGHD7* was identified using “table2hap()” function. In this step, accessions with heterozygotes or missing genotypes were eliminated. Finally, in line with previous studies [21], a total of 499 accessions and 8 main haplotypes were remained after elimination of rare haplotypes and missing genotypes.

**Fig. 3:**
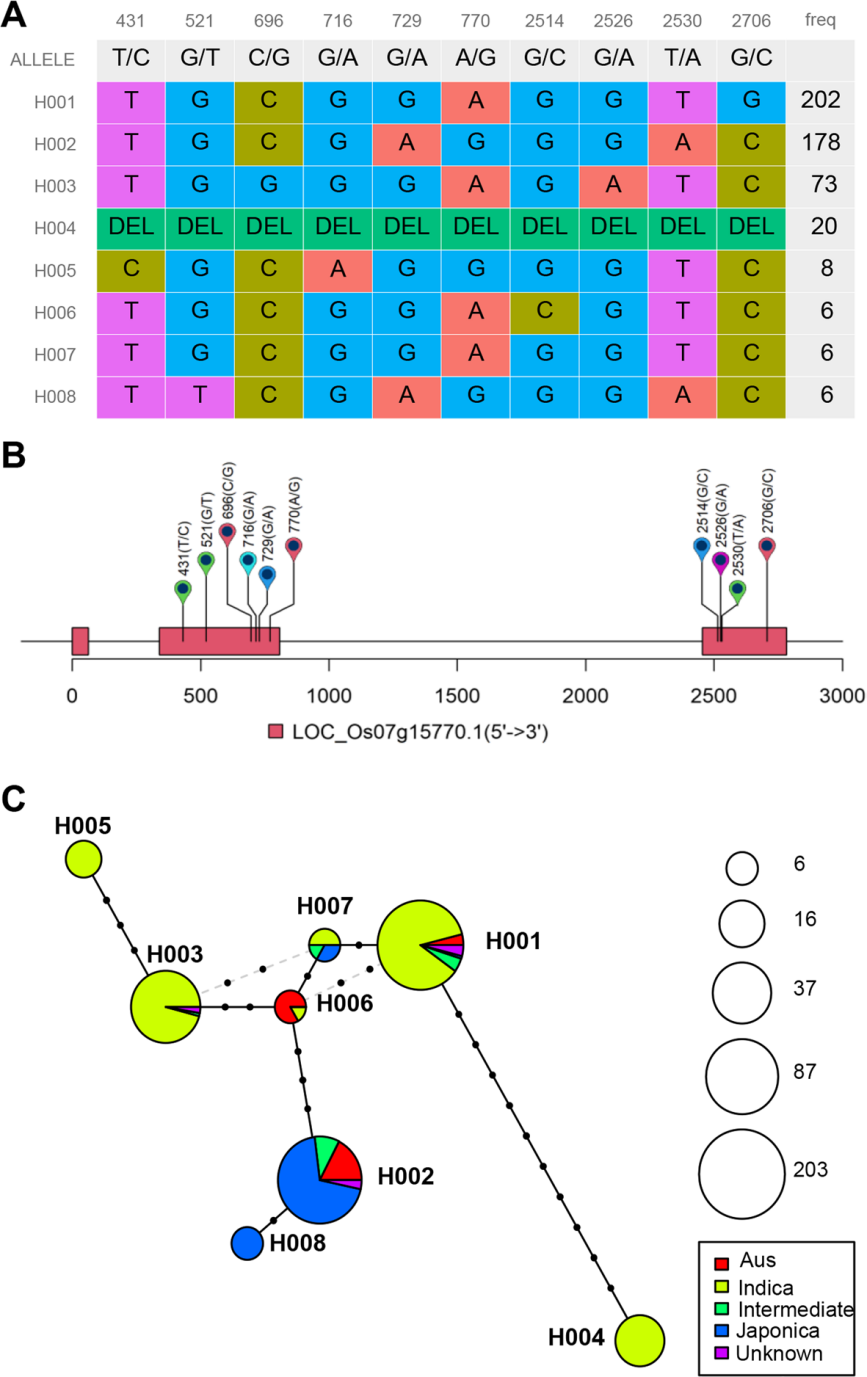
Visualization of haplotype classification, genomic variants and evolutionary network. **A** Haplotype classifications of *OsGHD7*, each line represent a haplotype and colored columns represents loci and frequency in the last column. **B** Visualization of variants position above gene model, the black line represents genome and rectangles represent exon. Flags represent variants and the coordinate with alleles in parenthesis were displayed above gene model. Two or more transcripts will be displayed in different colors. **C** Example of *OsGHD7* haplotype network. Each circle represents a haplotype and the size indicates accession number. The pies in different color represent the ratio of category in each haplotype. The symbols on line between haplotypes represent number of variants

The nucleotide coordinates were determined according to chromosome position in most variants database and next-generation sequencing results. But, it’s more meaningful if the coordinates of nucleotide initiated at the start codon (ATG) of target genes for further research. We then adjusted the coordinates of start codon to zero in haplotype results and annotation files by “hapSetATGas0()” and “gffSetATGas0()” function, respectively. The genotype of each haplotype was visualized using “plotHapTable()” function (Fig. 3A). Different nucleotide and Indels were assigned with different color, and the haplotype frequency was plotted at right. Moreover, variant details could be displayed bellow the ALLELE line. And then the position and allelic types of *OsGHD7* were visualized upon the gene schematic diagram (Fig. 3B) with “displayVarOnGeneModel()” function. Each box represents an exon, and the flag with position and genotype represents a variant.

The evolutionary relationship between haplotypes was visualized as a haplotype network (Fig. 3C). The variants between each closed haplotypes were marked as dots. Circle size represents the frequency of relevant haplotype and the pie angle represents proportion of corresponding group.

**Fig. 4:**
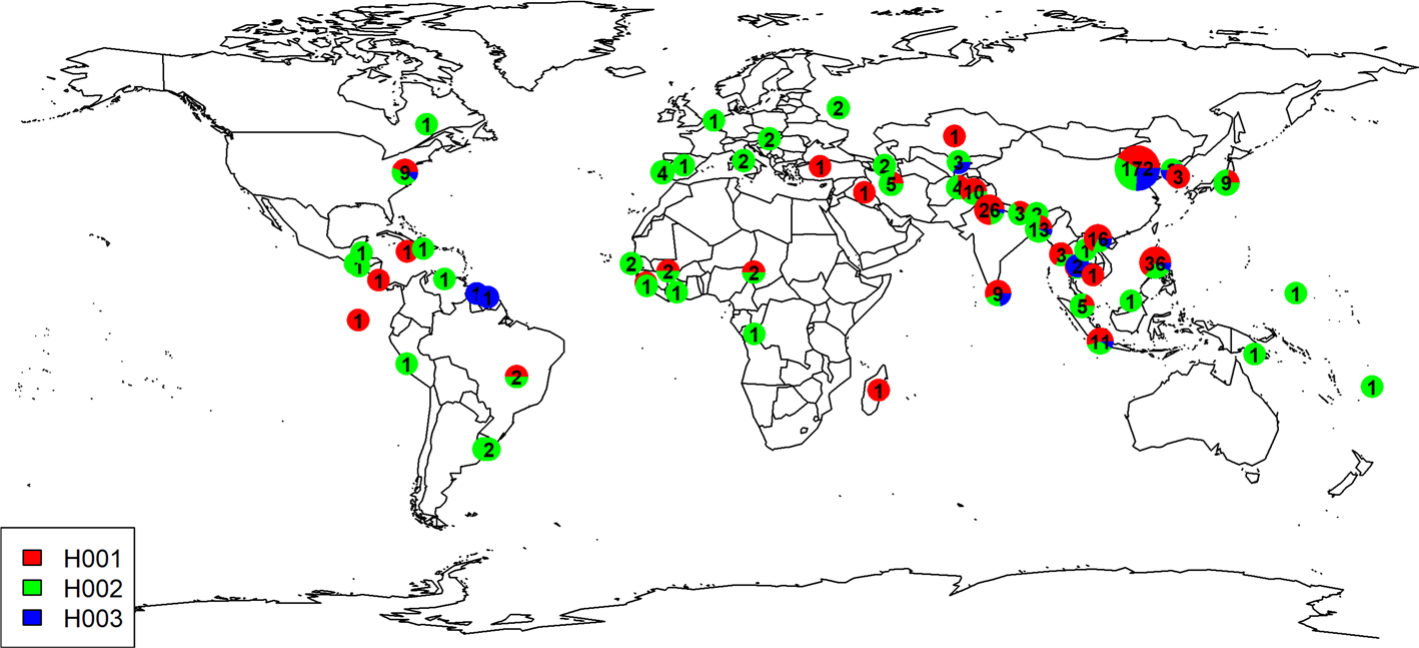
Example of geo-distribution of major haplotypes of *OsGHD7*. Circle size represents accessions counts and the pies in different color represent constituent ratios of classified haplotype categories for relevant accessions derived from different eco-regions. The Arabic number in circles indicate accession numbers at each location

**Fig. 5:**
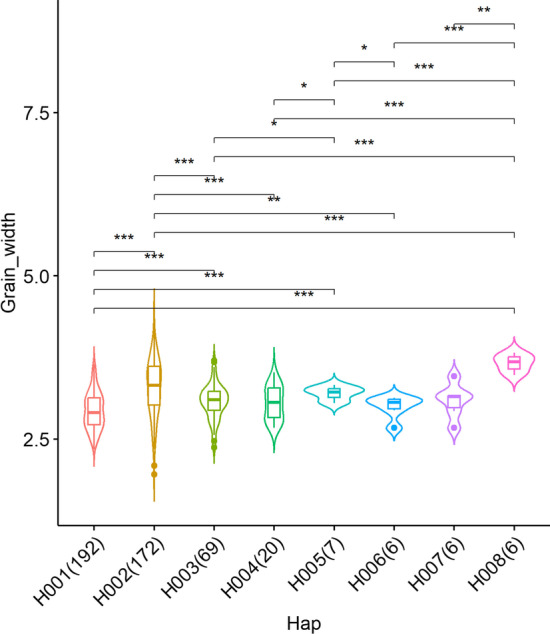
Example of trait comparison between haplotypes. Grain thickness comparisons among accessions carrying different haplotypes of *OsGHD7,* * indicates: *p* < 0.05, ** indicates: *p* < 0.01, *** indicates: *p* < 0.001

Global distributions of three main haplotypes in *OsGHD7* were illustrated in Fig. 4, and H003 is mainly distributed across Asia. Grain width of accessions carrying H001 and H002 were significantly lower and higher than others (Fig. 5), respectively. Therefore, the H002 haplotype would be preferred for breeding selections.

**Fig. 6:**
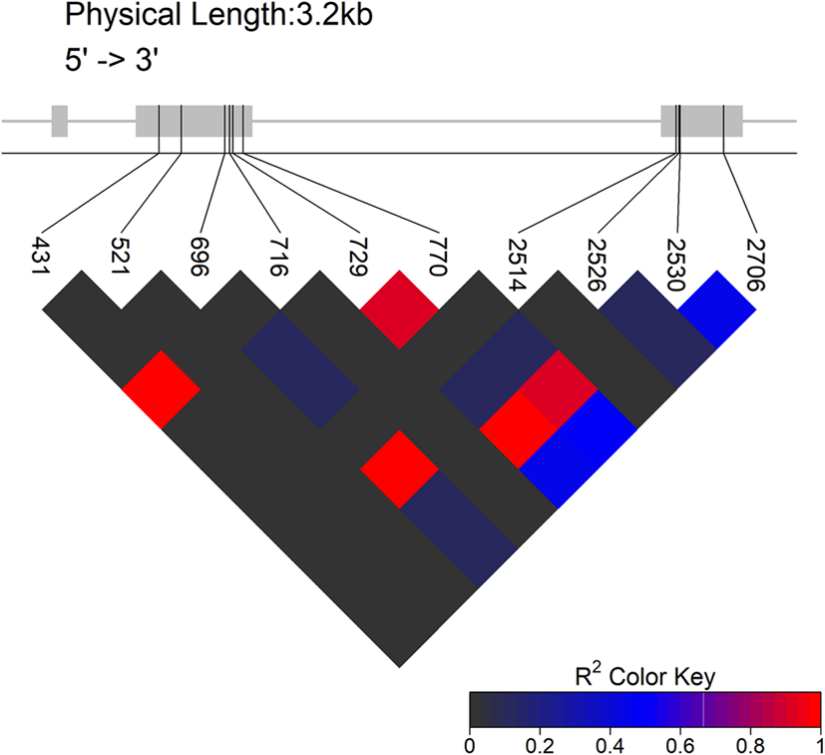
LD-block visualization of each site. The gene model was presented at top of the plot, the line represents genome and the rectangles represent exons. The oblique line bellows the gene model represent variants. The LD-block with colors key lie at the bottom

Linkage disequilibrium statistics were also performed and visualized with *hapResult* directly without format conversion through “plot_LDheatmap()” function. In *OsGHD7*, the 3' end variants were closely linked with each other compared with that of 5' end variant (Fig. 6).

## Discussion

During last two decades, many programs have been developed for haplotype analysis [25]. However, the limitation of current accessible programs is the lack of visualization and phenotypic effect results, which has restricted the application of superior haplotypes in breeding programs [26, 27]. Therefore, we tried to introduce geneHapR to facilitate further exploration of haplotypic variations.

Genotype data of target gene can be download from published variants database, or obtained from Sanger sequencing, or extract from variants calling results of next-generation sequencing project. And variants of thousands of individuals usually stored in large files. It’s a time-consuming work and challenge to extract variants with a Graphical User Interface program, such as: Excel, Notepad++ and EditPlus. The filterLarge*() functions in geneHapR provide a convenient way for researchers to efficiently extract variants from such large files on personal computer.

Publicly available database generated by genotyping microarrays and next-generation sequencing usually contain missing genotypes. Given that most individuals used in haplotype analysis were genetically unrelated, thus the genotype imputation algorism was not included in geneHapR. Furthermore, missing genotype often emerge due to low DNA quality, or inadequate genotype calling algorithms [28]. And heterozygous effects of allelic variants are usually unclear. Therefore, to assure the reliability of statistic results of interested haplotypes for target genes, individuals harboring missing genotype or heterogeneous sites were removed by default.

There are much more programs that can be used for haplotype identification, such as: HaploView and DnaSP. But the format of result data needs to be converted for downstream analysis. For instance, DNA sequences must be aligned and trimmed before haplotype identification using DnaSP, and result must be exported as “Roehl Data File” for haplotype network visualization using NetWork. To avoid format conversion works which is fallible and intricate, geneHapR provides a one-station solution and an easy-to-use toolkit for variants extraction, haplotype identification, statistics, and visualization. Comparison between geneHapR and other haplotype analysis software were summarized in Table 4, which revealed more comprehensive functions supported by geneHapR. For example, users can easily demonstrate genotypic variants, evolutionary relationships and main haplotypes’ geographical distributions after haplotype identification using geneHapR.

**Table 4:** Comparison between the geneHapR and other software

	geneHapR	CandiHap	Haplo.stats	HaploView	pegas	DnaSP
Variants table	Yes^a^	Yes	No^b^	No	No	Yes
Variants track	Yes	Yes	No	No	No	No
LD-block	Yes	Yes	No	Yes	No	No
Haplotype network	Yes	Yes	No	No	Yes	Yes^c^
Geographical distribution	Yes	No	No	No	No	No
Phenotype comparison	Yes	Yes	Yes	No	No	No

^a^“Yes” represents program supported this function

^b^“No” represents not supported

^c^Requires another software named Network

CandiHap is a recently released software that provides functions for haplotype identification, statistics, and visualization. Comparison between CandiHap and geneHapR was also conducted on the same platform of computer running windows 10 system with CPU Intel-i7-8700 (3.20 GHz) and 24 GB memory. In all comparisons, geneHapR shows a higher efficiency in haplotype identification process (Table 5). In geneHapR, the imported genotype data was converted into data.frame object, and haplotype identification was then directly conducted. While CandiHap needs three steps of data format conversion and the second one needs much more time. This might lead to the cost of geneHapR being hundreds of times faster than CandiHap.

**Table 5:** Average time cost comparison between geneHapR and CandiHap

	Variants	Individuals	geneHapR (s)	CandiHap (s)	Repeats
Comparison 1	1119	1844	8.713	2332.025	10
Comparison 2	586	1844	4.558	1254.409	20
Comparison 3	586	907	2.219	284.816	20
Comparison 4	868	406	0.429	81.803	20

To clarify the accuracy of geneHapR, we recalculated the haplotypes of *GHD7* with CandiHap. Accessions containing “DEL” were eliminated manually for compatibility. Finally, CandiHap identified seven haplotypes, harboring 202, 178, 73, 8, 6, 6 and 6 individuals, respectively, which is consistent with geneHapR. Furthermore, we also performed a comparison between geneHapR and DnaSP using genotype data of *SiTOC1* (Additional file 6), a functional gene controlling heading date in *Setaria italica* [29]. The haplotype result revealed by geneHapR is matchable with previously report [29] except the Indel size is one base pair longer and coordinates is one base pair smaller, due to the Indel identification algorithm is different in these two programs.

## Conclusion

The geneHapR package provides an easy-to-use toolkit for haplotype identification, statistics, phenotype association and result visualization analysis towards specific functional genes. The package has been submitted to CRAN and is available at: https://CRAN.R-project.org/package=geneHapR.

## Supplementary Information

**Additional file 1**: The R scripts used for  identification and visualization of *OsGHD7 *haplotypes.

**Additional file 2**: The genotype of *OsGHD7*.

**Additional file 3**: The annotation of *OsGHD7*.

**Additional file 4**: The phenotype of *OsGHD7*.

**Additional file 5**: The informaion of individuals used for *OsGHD7* haplotype identification.

**Additional file 6**: The genotype data of *SiTOC1 *in fasta format.

## Data Availability

The package is an open-source software released under the GPL-3 license, and it is freely available from CRAN (https://cran.r-project.org/package=geneHapR) and Gitee (https://gitee.com/zhangrenl/genehapr). The R script and all datasets used as examples during this study are included in supplementary information files. Project name: geneHapR. Project home page: https://gitee.com/zhangrenl/genehapr. Operating system(s): Windows, Linux, MacOS. Programming language: R. Other requirements: R >= 4.2. License: GNU GPL3. Any restrictions to use by non-academics: Not applicable.

## References

[1] Guo Z, Cao H, Zhao J, Bai S, Peng W, Li J (2022). A natural uORF variant confers phosphorus acquisition diversity in soybean. Nat Commun.

[2] Chen J, Upadhyaya N, Ortiz D, Sperschneider J, Li F, Bouton C (2017). Loss of AvrSr50 by somatic exchange in stem rust leads to virulence for Sr50 resistance in wheat. Science.

[3] Sobreira D, Joslin A, Zhang Q, Williamson I, Hansen G, Farris K (2021). Extensive pleiotropism and allelic heterogeneity mediate metabolic effects of IRX3 and IRX5. Science.

[4] Trujillo C, Rice E, Schaefer N, Chaim I, Wheeler E, Madrigal A (2021). Reintroduction of the archaic variant of NOVA1 in cortical organoids alters neurodevelopment. Science.

[5] Huang Y, Wang H, Zhu Y, Huang X, Li S, Wu X (2022). THP9 enhances seed protein content and nitrogen-use efficiency in maize. Nature.

[6] Nagai K, Mori Y, Ishikawa S, Furuta T, Gamuyao R, Niimi Y (2020). Antagonistic regulation of the gibberellic acid response during stem growth in rice. Nature.

[7] Sasani T, Ashbrook D, Beichman A, Lu L, Palmer A, Williams R (2022). A natural mutator allele shapes mutation spectrum variation in mice. Nature.

[8] Abell N, DeGorter M, Gloudemans M, Greenwald E, Smith K, He Z, Montgomery S (2022). Multiple causal variants underlie genetic associations in humans. Science.

[9] Tcw J, Qian L, Pipalia N, Chao M, Liang S, Shi Y (2022). Cholesterol and matrisome pathways dysregulated in astrocytes and microglia. Cell.

[10] Paradis E (2010). pegas: an R package for population genetics with an integrated-modular approach. Bioinformatics.

[11] Rozas J, Ferrer-Mata A, Sánchez-DelBarrio JC, Guirao-Rico S, Librado P, Ramos-Onsins SE (2017). DnaSP 6: DNA sequence polymorphism analysis of large datasets. Mol Biol Evol.

[12] Li X-K, Shi Z-Y, Gao J-H, Wang X-C, Guo K (2023). CandiHap: a haplotype analysis toolkit for natural variation study. Mol Breeding.

[13] Barrett J, Fry B, Maller J, Daly M (2005). Haploview: analysis and visualization of LD and haplotype maps. Bioinformatics.

[14] Sinha P, Singh VK, Saxena RK, Khan AW, Abbai R, Chitikineni A (2020). Superior haplotypes for haplotype-based breeding for drought tolerance in pigeonpea (*Cajanus cajan* L.). Plant Biotechnol J.

[15] Lin M, Griessenauer CJ, Starke RM, Tubbs RS, Shoja MM, Foreman PM (2019). Haplotype analysis of SERPINE1 gene: Risk for aneurysmal subarachnoid hemorrhage and clinical outcomes. Mol Genet Genomic Med.

[16] Ito K, Murphy D (2013). Application of *ggplot2* to pharmacometric graphics. CPT Pharmacomet Syst Pharmacol.

[17] The Plant Genomics Resource. https://phytozome.jgi.doe.gov. Accessed 7 Feb 2023

[18] Knaus BJ, Grünwald NJ (2017). VCFR: a package to manipulate and visualize variant call format data in R. Mol Ecol Resour.

[19] Michael L, Robert G, Vincent C (2009). rtracklayer: an R package for interfacing with genome browsers. Bioinformatics.

[20] Xue W, Xing Y, Weng X, Zhao Y, Tang W, Wang L (2008). Natural variation in *Ghd7* is an important regulator of heading date and yield potential in rice. Nat Genet.

[21] Liu R, Feng Q, Li P, Lou G, Chen G, Jiang H (2022). *GLW7.1*, a strong functional allele of *Ghd7*, enhances grain size in rice. Int J Mol Sci.

[22] Zhao H, Yao W, Ouyang Y, Yang W, Wang G, Lian X (2015). RiceVarMap: a comprehensive database of rice genomic variations. Nucleic Acids Res.

[23] Wang C, Yu H, Huang J, Wang W, Faruquee M, Zhang F (2020). Towards a deeper haplotype mining of complex traits in rice with RFGB v2.0. Plant Biotechnol J.

[24] Kawahara Y, de la Bastide M, Hamilton JP, Kanamori H, McCombie WR, Ouyang S (2013). Improvement of the *Oryza sativa* Nipponbare reference genome using next generation sequence and optical map data. Rice.

[25] Li S, Zhang Y, Fan C, Chen Y, Deng C, Hu Z (2018). Advances in haplotype analysis technique. Sheng Wu Gong Cheng Xue Bao.

[26] Collard B-C, Mackill D-J (2008). Marker-assisted selection: an approach for precision plant breeding in the twenty-first century. Philos Trans R Soc Lond B Biol Sci.

[27] Oladosu Y, Rafii MY, Samuel C, Fatai A, Magaji U, Kareem I (2019). Drought resistance in rice from conventional to molecular breeding: a review. Int J Mol Sci.

[28] Marchini J, Howie B, Myers S, McVean G, Donnelly P (2007). A new multipoint method for genome-wide association studies by imputation of genotypes. Nat Genet.

[29] Zhang L, Zhi H, Tang S, Zhang R, Zhang W, Jia G (2021). Characterizations of transcriptional and haplotypic variations of SiTOC1 in foxtail millet. Sci Agric Sin.

